# Distributed video coding for wireless video sensor networks: a review of the state-of-the-art architectures

**DOI:** 10.1186/s40064-015-1300-4

**Published:** 2015-09-17

**Authors:** Noreen Imran, Boon-Chong Seet, A. C. M. Fong

**Affiliations:** School of Engineering, Auckland University of Technology, Auckland, New Zealand; School of Computing Science, University of Glasgow, Glasgow, UK

**Keywords:** Distributed video coding, Wireless video sensor networks, PRISM, Pixel domain Wyner–Ziv, Transform domain Wyner–Ziv

## Abstract

Distributed video coding (DVC) is a relatively new video coding architecture originated from two fundamental theorems namely, Slepian–Wolf and Wyner–Ziv. Recent research developments have made DVC attractive for applications in the emerging domain of wireless video sensor networks (WVSNs). This paper reviews the state-of-the-art DVC architectures with a focus on understanding their opportunities and gaps in addressing the operational requirements and application needs of WVSNs.

## Background

Rapid advancements in computing hardware and data communication have provided a platform to develop and deploy several innovative services and systems. The concept of spatially distributed battery powered sensors that cooperatively deliver their sensed information using radio channels to some common location has eventually taken the form of a wireless sensor network (WSN). Such a networked sensing infrastructure can enable a wide spectrum of applications ranging from military systems with advanced scouting and surveillance capabilities to civilian systems for environment and health monitoring, person locator services, detection of malfunctioning machinery in industrial plants, and inventory control.

**Fig. 1:**
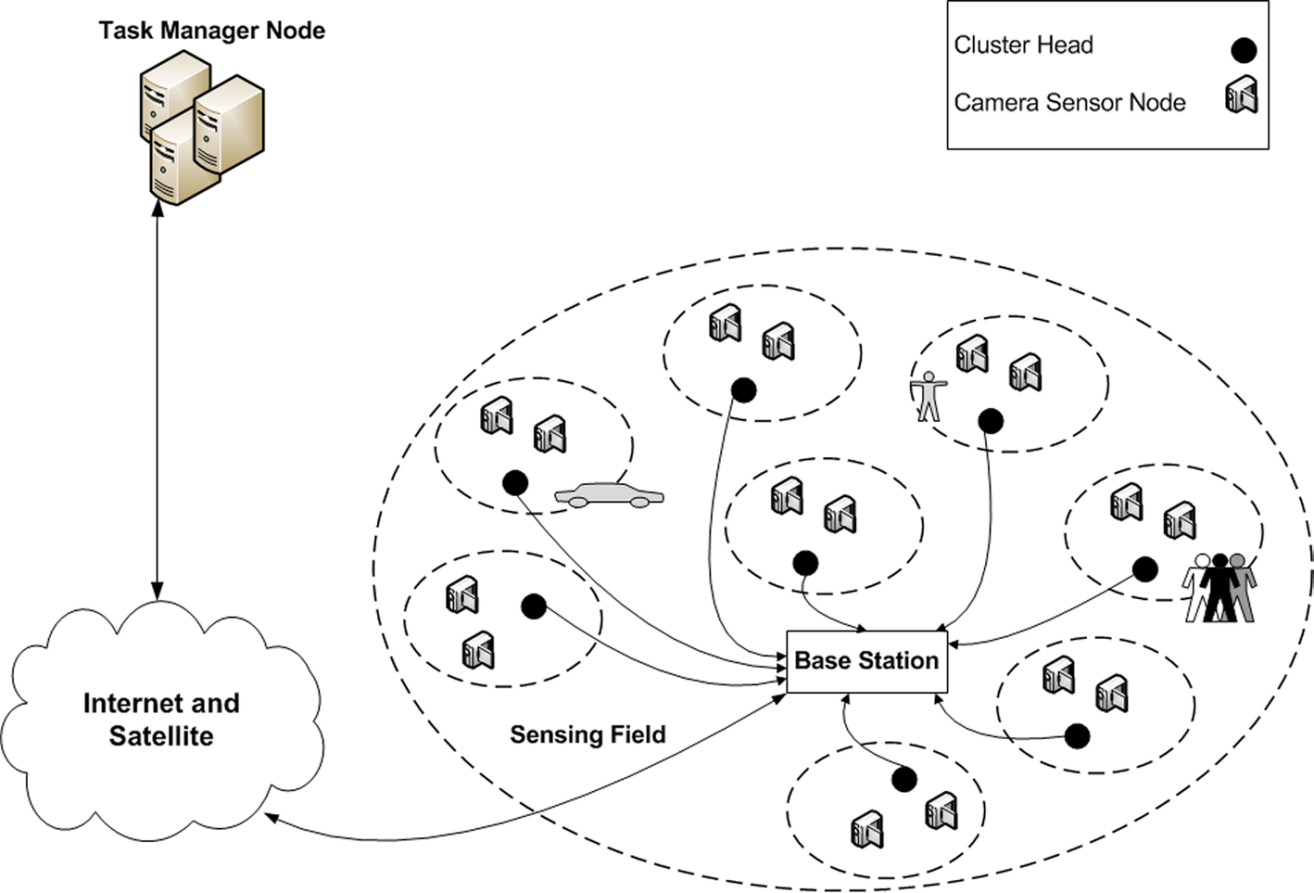
WVSN architecture

Recently, wireless video sensor networks (WVSN) and their associated theoretical and practical challenges have drawn significant attention from the research community. Such growing interest can be attributed to new applications enabled by large-scale networks of small camera devices capable of capturing visual information from their surrounding environments, performing simple processing/compression on the captured data, and transmitting it to remote locations (e.g. base stations) as shown in Fig. 1. Today, multifunctional wireless sensors that gather scalar as well as audio-visual data are used in various applications, including emergency response and health-care monitoring, where multimedia information (particularly video) is indispensable.

Video compression is a relatively complex operation that can consume significant processing and energy resources in resource-limited camera sensor nodes. Conventional compression standards are computation intensive but perform very well when complexity of the encoder is not a major concern. DVC is an emerging video coding paradigm for applications with limited resources available at encoder. It reverses the conventional video coding paradigm by shifting the encoder’s complexity entirely or partially to the decoder, which is assumed to be more resourceful than the encoder (Puri et al. 2006). Therefore, DVC based encoders are much simpler, and a number of different DVC architectures (e.g. Aaron et al. 2004a; Ascenso and Pereira 2007; Kubasov et al. 2007a) have been proposed in literature.

The conventional video coding architecture has been challenged by the emergence of WVSNs. Traditional state-of-the-art video coding standards such as H.264 (Kalva 2006), MPEGx (Le Gall 1991) are pertinent to the broader class of applications that support encoders with complexity of at least 5–10 times greater than that of the decoder (Dufaux et al. 2009). These video coding architectures suit applications such as streaming video-on-demand (VoD), video broadcasting, digital home systems, and multimedia collaboration that requires video to be encoded once and decoded several times by consumers (Pereira et al. 2008; Melodia and Akyildiz 2011).

Conventional video coding architectures are primarily based on hybrid discrete cosine transformation (DCT) and interframe predictive video coding (PVC) frameworks. These frameworks allocate codec functionalities such that most of the high complexity operations that involve exploiting spatial and temporal correlation, e.g. motion estimation and compensation, are executed at the encoder, while the decoder performs lower complexity operations such as entropy decoding, frame prediction, inverse quantization, and DCT on the bitstream received from encoder (Hsia et al. 2013; Kim et al. 2014).

A video sequence is a time-indexed set of frames (images) which contains a high degree of temporal redundancy among adjacent frames. Each frame is divided into sub-blocks for coding purpose. Conventional video coding employs two primary coding modes (Schwarz et al. 2007):

- *Inter*-*frame coding mode* Compression in inter-frame coding mode exploits not only temporal but also spatial correlation among video frames and performs high complexity motion estimation and compensation operations to predict the best matching block for the block under reconstruction. Only the residue between the given block and the corresponding predictor is encoded and transmitted. Therefore, compression efficiency of inter-frame coding mode is very high at the expense of higher computational complexity and less robustness against packet losses.
- *Intra*-*frame coding mode* In contrast, intra-frame coding mode only exploits the spatial correlation for encoding a block in a given frame. Therefore, the encoding complexity and compression efficiency is lower than inter-frame coding mode. However, intra-frame coding mode does not depend on adjacent frames and is more robust against packet losses since it treats each frame as a still image and encodes it separately without exploiting dependencies between adjacent frames.

Irrespective of the coding modes, the PVC architecture has two significant drawbacks (Girod et al. 2005; Pereira et al. 2008):

- *Rigid allocation of functionality* PVC enforces rigid allocation of functionality between encoder (complex) and decoder (simple), with the complex motion search operation dominating the overall encoder’s complexity.
- *Drift error* Since no real-time synchronization channel exists between PVC encoder and decoder, there is a higher probability of prediction mismatch in uncontrolled environment such as communication over wireless/radio links which are more prone to channel errors.

As opposed to conventional video coding that follows *joint*-*encoding/independent*-*decoding* configuration, DVC architecture follows an *independent*-*encoding/joint*-*decoding* configuration which is more feasible for video applications in WVSNs. DVC reverses the conventional structure of the video codec by exploiting source statistics at the decoder.

The theory of DVC stipulates that the source statistics of two or more correlated video sequences can (wholly or partially) be exploited at decoder given that the bitstreams received from multiple encoders performs joint decoding via analyzing statistical correlation between them. It should also be noted that the term *distributed* in DVC originally refers to the encoding mode rather than the encoder‘s physical location (Girod et al. 2005; Dufaux et al. 2009).

The shift of complexity in DVC is accomplished by assigning to the decoder the responsibility for generating the prediction, and relieving the encoder from such complex and computation intensive task. However, the encoder still possesses the ability to select the best prediction based on a comparison with the original frame to be coded. On the other hand, the decoder is unable to perform such comparison since it has only access to already decoded information and not the original frame, which complicates the decoder’s task to estimate a precise motion field as compared to conventional predictive video coding.

To reliably deliver high quality video to the sink server (decoder) in energy-constrained WVSN, the use of energy-efficient video coding scheme, rate-control and QoS management algorithms is critical. With a competitive compression performance and lower energy consumption, DVC is a promising alternative to traditional PVC architectures which are not suitable for WVSNs.

In this paper, a comprehensive review of the state-of-the-art on DVC based architectures appropriate for WVSN is presented for the first time. The review includes a comparative discussion of several well-known architectures in literature with a focus on their functional aspects, performance comparison and suggests a number of possible enhancements to the design of these architectures.

The rest of the paper is organized as follows: “Wireless video sensor network: challenges and issues” presents a general overview of WVSN and its current challenges. “Information theory of PVC and DVC” presents the information theory underpinning the conventional (PVC) and DVC architectures, “DVC in wireless sensor networks” discusses the significance of DVC architecture in the context of WSNs, “DVC architectures” overviews representative DVC architectures, “Comparison and analysis” compares and analyzes key differences between the architectures discussed in “DVC architectures”, and finally “Conclusion” concludes the paper.

## Wireless video sensor network: challenges and issues

A WVSN is a network of spatially distributed sensor nodes, each equipped with a miniaturized camera that captures, compresses, and transmits visual information (image/video) about its surroundings to a sink node or base-station for further content analysis and distribution. The foundation of WVSNs can be understood as the convergence between the concepts of WSNs and distributed smart cameras, i.e. it encompasses technologies from various disciplines such as wireless communications and networking, signal processing, security, computer vision, control and robotics (Harjito and Song 2010).

The self-organizing, flexible, and scalable characteristic of WVSN is one key factor for its widespread popularity. Unlike WSNs where sensor nodes capture and transmit only simple scalar data such as temperature, pressure, and humidity, multimedia data is based on rich streaming media generated at a higher rate, and thus requires more complex processing, memory storage, higher network bandwidth, and energy for transmission. At the same time, WVSNs have to deal with optimization of performance parameters such as delay, throughput, network lifetime, and quality of service (QoS).

## Information theory of PVC and DVC

*Problem definition* Assume *X* and *Y* are two statistically correlated, independently and identically distributed (i.i.d) video sequences from two separate encoders that are aware of the existence of each other. Moreover, the decoder has complete information about the encoders. The problem is to determine the minimum encoding (bit) rate for each of the video sources such that their joint decoding at the decoder reconstructs each of the video sequence with sufficient accuracy. This problem can be addressed using joint entropy, since video sequences *X* and *Y* are statistically correlated. Two different methods to reconstruct them are as follows:

### PVC methodology: joint-encoder, joint-decoder

If the two statistically dependent video sequences *X* and *Y* are encoded together to exploit their statistical dependencies, the minimum lossless rate is *H*(*X*,*Y*), which represents their joint entropy

1 $$R_{(X,Y)} = H\left( {X,Y} \right).$$

### DVC methodology: independent-encoder, joint-decoder

However, if the video sequences *X* and *Y* are encoded independently, their respective encoding rate is:

2 $$R_{X } \ge H\left( X \right)$$

and

3 $$R_{Y} \ge H\left( Y \right)$$

where *H*(*X*), and *H*(*Y*), represents the entropy^1^ of *X*, and *Y*, respectively.

Then the required encoding rate is given by:

4 $$R_{X } + R_{Y} \ge R_{(X,Y)} .$$

**Fig. 2:**
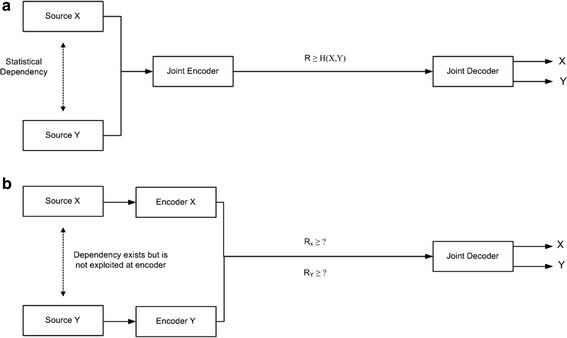
Video coding architecture: **a** PVC methodology; **b** DVC methodology

The functional block diagram of PVC and DVC architectures is shown in Fig. 2a, b, respectively. One may consider whether if it is possible to reconstruct the video sequence with small error probability at encoding rates lower than individual entropies *H*(*X*) and *H*(*Y*). Distributed source (video) coding provides an answer to this problem as follows:

*Slepian*–*Wolf theorem for lossless compression* Assume that the minimum encoding rate for lossless reconstruction of signal $X$ is equal to the signal entropy given by *H*(*X*). To determine the lossless encoding rate between two (or more) related source signals *X* and *Y*, exploit the statistical correlation between these signals and encode them jointly with the joint signal entropy of $H(X,Y)$.

The Slepian–Wolf theorem (Slepian and Wolf 1973) stipulates that for lossless reconstruction of signals, the encoding rate similar to the one employed in joint encoding of signals *X* and *Y*, can be attained even if *X* and *Y* are encoded independently (neither *X* nor *Y* has access to each other), given that they will be jointly reconstructed at the decoder with an arbitrarily small error. The following set of equations represents the individual as well as joint encoding rates of signals *X* and *Y*:

5 $$R_{X} \ge H\left( {X|Y} \right)$$

6 $$R_{Y} \ge H\left( {Y|X} \right)$$

7 $$R_{X} + R_{Y} \ge H\left( {X,Y} \right).$$

In practice, the coding performance is determined by the capacity of the correlation channel that approaches the Slepian–Wolf bound used by sophisticated turbo or low-density parity check (LDPC) codes.

**Fig. 3:**
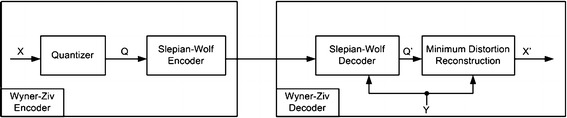
Wyner–Ziv logical architecture (Girod et al. 2005)

*Wyner*–*Ziv theorem for lossy compression* Wyner and Ziv (1976) proposed an extension to Slepian–Wolf theorem by defining the same scenario (as discussed above) of independent encoding but in the context of lossy compression. It states that for statistically correlated signals *X* and *Y*, if encoding of *Y* has been performed at the rate $H(Y)$, then for joint reconstruction at decoder, only the minimum encoding rate of *X* needs to be determined with a certain upper bound on the distortion *D* in the reconstructed signal. Here, *Y* acts as side information to estimate *X*, and the distortion function provides the minimum encoding rate $( R_{X} )$ for reconstruction of $X$. Wyner–Ziv theorem is also widely known as Wyner–Ziv rate-distortion distributed video coding theorem, and its logical framework is shown in Fig. 3.

Considering the requirements of video coding for real-time applications, the Wyner–Ziv approach of encoding signals is more realistic and appropriate (Tomé 2009) since it accommodates a certain level of distortion (such as packet losses) during signal reconstruction which is likely in real-time wireless transmission.

## DVC in wireless sensor networks

Wireless sensor networks (WSNs) have gained considerable attention in recent years due to their vast application domain, flexible deployment structure, and most of all, the availability of low–cost CMOS sensor modules. The application domain for WSN spans from surveillance monitoring to healthcare, traffic and industrial control sectors (Yang 2014). The self-organizing, flexible, and easily scalable infrastructure of WSN is one key factor for its widespread popularity. On the other hand, WSNs have to deal with optimization of performance parameters such as delay, throughput, network lifetime, and quality of service (QoS) (Magaiaa et al. 2015). Moreover, WSN-based multimedia applications have limited bandwidth which necessitates the need for more efficient compression algorithms. Video streams are highly bandwidth demanding. Transmitting these streams to the sink via several intermediate nodes over radio links requires much higher bandwidth than that required for transmitting scalar data (Imran et al. 2012). The high bit rate and multi-hop transmission make the system prone to congestion due to both intra- and inter-flow interferences. The congestion becomes more serious when there are multiple flows and where traffic exhibits a many-to-one pattern (Liang 2011).

Different WSN applications may have different requirements in terms of bandwidth, processing and compression, among others. Video streaming involves continuous capturing and delivery of data that requires optimized encoding and compression algorithms in addition to efficient hardware to meet the often demanding QoS requirements (Akyildiz et al. 2007). In the context of WSNs, the approaches for network layer QoS can be based on reliability or timeliness of video delivery (Misra et al. 2008). For example, some applications are delay-tolerant, but require reliable and error-free data transmission. It involves packet retransmissions and multipath routing through which a sensor node can inject multiple copies of same packet into different paths so that at least one copy is able to make it to the sink.

In this section, we also discuss the applicability of DVC in WSNs and how well it fits under its constrained environment. Almost all of the video coding applications fall within the two classes of application models, namely *downlink* and *uplink* models.

The downlink application model is associated with the broadcasting approach, where low-complexity decoder is desirable and the complexity of the encoder is not an issue. The encoder of the downlink application model is more like a base-station that does not have computational constraints. Applications such as video streaming, broadcasting, and telephony are belonging to the downlink application model.

On the other hand, the uplink application model, also known as wireless video model, represents the reverse architecture, where low-complexity encoder is required and the complexity of the decoder is not a major concern. Consider an environment which comprises of several integrated units (wireless video sensors) that include video sensing modules with on-board processing and transmission functionality. These integrated units are interconnected with each other via wireless networking protocol, communicate over radio links, and have limited battery life. They are used to capture, compress and transmit compressed video data from their surroundings to a centralize location (base-station) over single- or multi-hop environment. Applications such as mobile video calling, wireless video surveillance, and monitoring are belonging to the uplink application model (Alnuaimi et al. 2011; Elamin et al. 2012; Puri et al. 2006; Xue et al. 2008).

Popular video coding standards such as MPEGx and H.264/AVC supports only the downlink application model, while the DVC is a solution for applications of the uplink model. Using DVC in uplink application domain has potential advantages such as flexible allocation of functionality from encoder to decoder (and even transcoder in some cases), low-encoding complexity that in turn leads to low-power consumption, longer battery life, and light-weight processing devices (Pereira et al. 2008).

DVC theory introduces a notion of shifting computational complexity from encoder to decoder, which makes it a viable option for applications in WSN domain where encoders are resource-limited wireless sensor nodes, and decoder is a more powerful base-station. The rate-distortion performance of DVC codecs is also comparable to that of conventional H.264/AVC Intra-frame coding (Artigas et al. 2007). Another important feature of DVC which is desirable in WSNs is that it provides better error resilience. DVC does not employ any prediction loops at encoder and therefore no estimation and prediction errors are sent to the decoder. Rather, the side information module in DVC decoder predicts the frame by exploiting statistical and temporal correlation among video frames (Pereira et al. 2008).

Exploitation of correlation in multi-view camera architecture is also a distinguished feature of DVC for WSNs. For example, if multiple wireless video sensors have some overlapping coverage areas, they may not need to communicate with each other to exploit that correlation; rather they encode their video sequences independently and proceed with the transmission to the decoder, which is responsible for processing the correlation among video sequences from these sources (Xue et al. 2008).

**Fig. 4:**
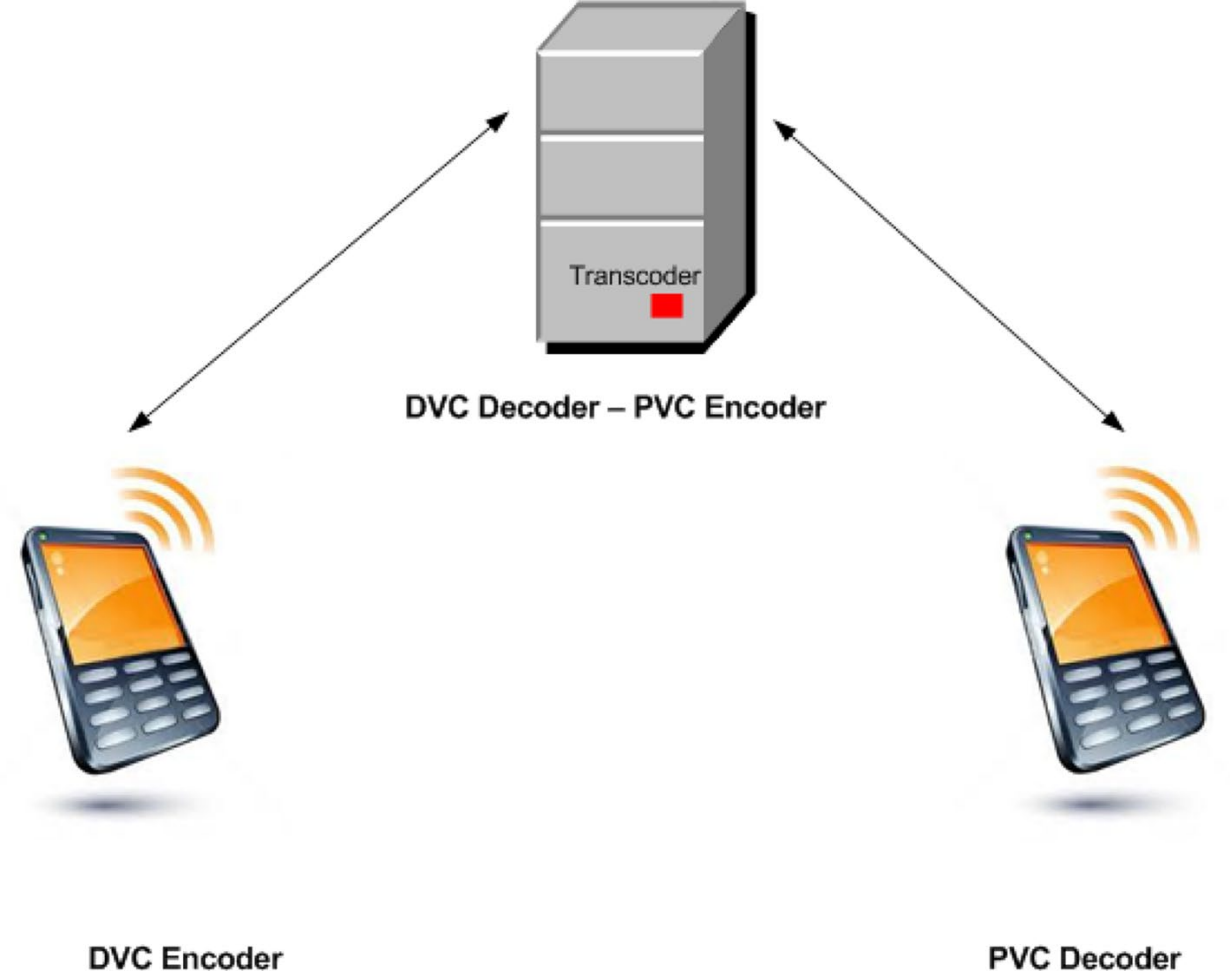
Video transcoder framework

DVC exhibits higher computational complexity at decoder, which consequently makes it a less viable option for applications that requires real-time decoding in WSN. By itself, DVC is not sufficient to support an end-to-end wireless video surveillance system. For this purpose, the use of transcoder is mandatory to simplify both transmitter (encoding node) and receiver (decoding node) sides of the network. However, the middle tier, i.e. the transcoder, requires video to be encoded by conventional PVC based codec. The basic architecture of transcoder is shown in Fig. 4.

## DVC architectures

As discussed in “Information theory of PVC and DVC”, it is theoretically possible to achieve a coding efficiency comparable to that of conventional PVC paradigm when two sources encoded their correlated video sequences independently and decoded them jointly by exploiting the statistical correlation. However, the theorems have not identified the methodology to attain practically the same compression efficiency. Hence, researchers have devised their own implementation of DVC codecs with various different side information generation methods. For example, some codecs use the same DVC encoder while others employ the conventional PVC encoders (Intra mode) to generate side information. Practically, the compression efficiency of DVC codecs is comparable to PVC codecs (executing in Intra-mode) (Pereira et al. 2008).

Two primary DVC approaches have been proposed in literature, namely Berkeley (Puri and Ramchandran 2002) and Stanford (Girod et al. 2005; Aaron et al. 2002, 2004b) video coding architectures. The Berkeley architecture known as PRISM followed a *block*-*based* encoding approach with motion estimation module at decoder. On the other hand, the Stanford architecture adopted a *frame*-*based* encoding approach, which has gained much popularity because of its comparatively better rate-distortion performance. Subsequently, enhancements were made to the original design such as the extension of pixel domain Wyner–Ziv (PDWZ) to transform domain Wyner–Ziv (TDWZ), replacement of turbo codes by LDPC channel codes, development of more efficient reconstruction algorithms, and employment of Intra-frame coding mode from state-of-the-art PVC architectures for more efficient generation of side information. In the following section, we discuss the three main DVC architectures as mentioned above in more detail, namely PRISM (Puri and Ramchandran 2002), PDWZ (Aaron et al. 2002), and TDWZ (Aaron et al. 2002, 2004b) codec.

### Power-efficient, robust, high-compression, syndrome-based multimedia coding (PRISM) architecture

**Fig. 5:**
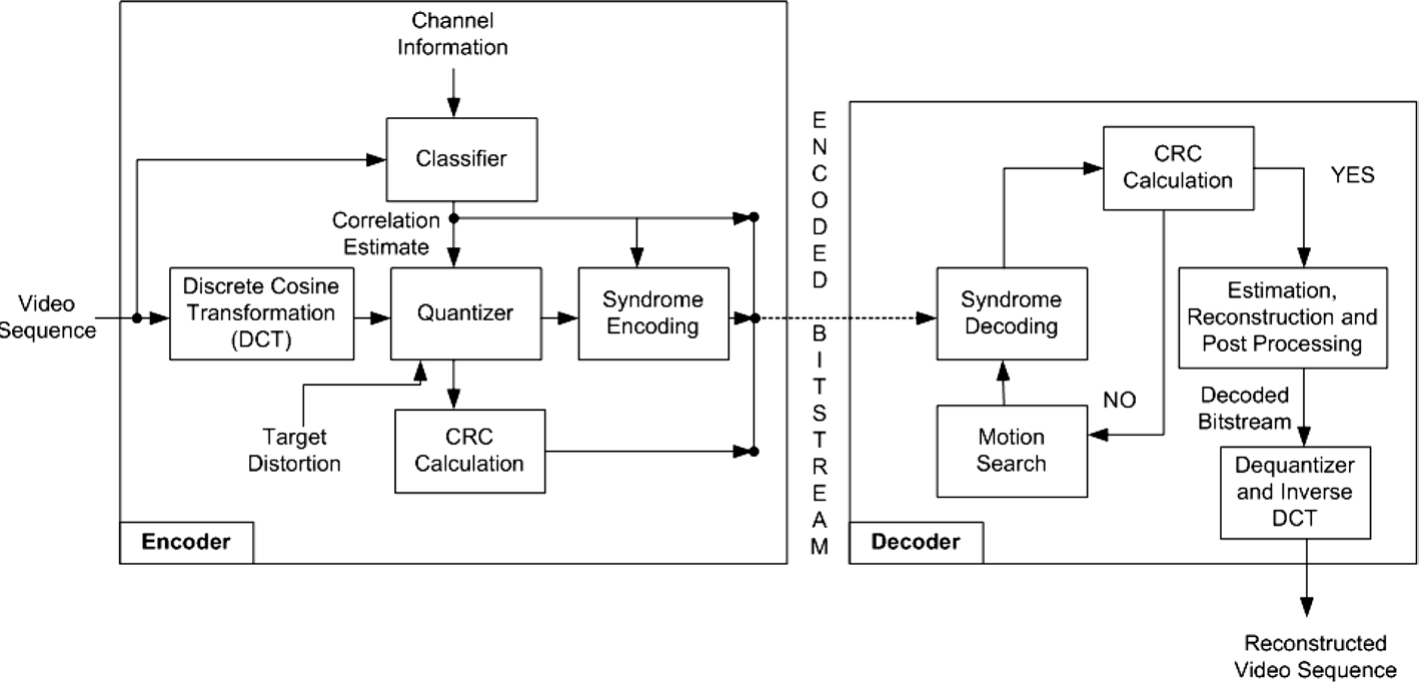
PRISM architecture

The PRISM (also known as Berkeley DVC) architecture proposed by Puri and Ramchandran (2002) was designed to achieve compression efficiency comparable to that of PVC but with a lower encoding complexity (Fig. 5). PRISM was introduced as a first step towards the design of an uplink-friendly video coding architecture. Unlike Wyner–Ziv framework (side-information based video codec), PRISM is based on a different side information paradigm where there is an inherent uncertainty in the state of the side information. Such side information paradigm allows the expensive motion compensation task to be shifted from the encoder to the decoder while exploiting the temporal redundancies of a video sequence. The primary constituent modules of PRISM encoder and decoder are as follows:

#### Encoder

Each video frame is first decomposed into non-overlapping blocks of size $n \times n$ where $n \in \{ 8, 16\}$. Thereafter, each macro-block undergoes the following encoding steps.

*Transform coding* 2D discrete cosine transform (DCT) is employed to transform each macroblock from spatial to frequency domain with a computational complexity equivalent to that of performing Intra-frame coding. The transformation yields DCT coefficients for each of the macroblock that must be transformed into quantized codewords prior to encoding.

**Fig. 6:**
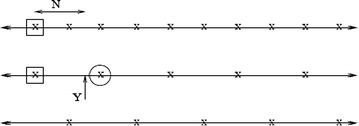
Scalar quantization (Natário et al. 2005)

*Scalar quantization* Let $X$ represents the current macroblock to be encoded, *Y* to be its side information generated from a previously reconstructed frame, and *N* is the correlation noise. In Fig. 6, the first line represents the quantized codewords for *X* while the next two lines represent the corresponding partitions of the quantized codeword space of *X*. The box shows the observed codeword which exists in the first partition. If the quantization step is less than the magnitude of $N$, and the decoder decodes the circled codeword (side information), this will lead to a decoding error. Therefore, the choice of quantization step should be directly proportional to the standard deviation of *N*. The scalar quantization block generates the space for evenly distributing the quantized codewords for $X$.

*Syndrome encoding* Following the quantization step, the quantized codewords are partitioned using Euclidean space Trellis channel code which operates on BCH^2^ code (Bose and Ray-Chaudhuri 1960) for error correction. A lightweight convolution process is executed between the parity matrix of Trellis channel code and quantized codewords to generate coset index (syndrome) for each codeword.

*Refine quantization* In scalar quantization, the step size is lower bounded by the correlation noise *N*, which is crucial to avoid decoding errors (Fig. 6). Depending on the target reconstruction quality, step size is chosen to be fine or coarse. Therefore, the refined quantization step is applied to coefficients to fine-tune codewords such that the desired target quality is achieved.

**Fig. 7:**
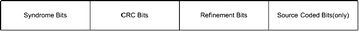
PRISM’s bitstream packet format (Puri and Kannan 2003)

*CRC calculation* The original PRISM architecture was later extended to include an error checking module at encoder site that improves the overall reconstruction quality: It was observed in (Puri and Ramchandran 2002) that side information encoding is performed in relation to the motion estimation and prediction error. The latter represents the mismatch between the current block that underwent encoding and its best predictor from frame memory. This implies that reconstruction of source signal is significantly dependent on the frame memory available at decoder. Therefore, an error checking module was incorporated in (Puri and Kannan 2003) that computes and transmits cyclic redundancy check (CRC) bits of quantized sequence along with the sequence itself. The bitstream format of the associated block is shown in Fig. 7.

#### Decoder

*Syndrome decoding* The syndrome bits of a sequence received at decoder are aligned according to trellis channel code to generate the quantized codeword sequence. Thereafter, the decoder employs Viterbi algorithm (Forney 1988) to determine the closest predictor sequence among the set of predictor sequences.

*Motion search and CRC calculation* While performing motion search on decoded syndrome bits, the decoder looks up the entire set of predictors for the best match and compares the bit sequence with the corresponding CRC check sequence, and marks it as a successful reconstruction if an exact match occurs between the two.

*Motion estimation, reconstruction and post processing* The quantized codeword sequence together with the predictor is used for the reconstruction (transform coefficients) of the source sequence. The PRISM framework can adopt any of the reconstruction algorithms from spatio-temporal correlation interpolation to efficient extrapolation algorithms.

*De*-*quantizer and inverse transformation* Following the reconstruction of the transform coefficients of the predicted source signal, de-quantization and inverse DCT transformation is performed to extract pixel values of the block.

#### Enhancements to PRISM

In (Majumdar and Ramchandran 2004), multi-level coset codes for PRISM architecture are presented. The codebook for each level is computed by coding bit at that level $B_{i}$ and the remaining (*i* − 1) bits are determined by the bits at previous levels $B_{j}$ (where $B$, *i* and *j* represents the coding bit, current coset level, and previous coset level, respectively, for 1 ≤ *j* ≤ *i* − 1). Therefore, quantization of *X* to the closest coset is done at the root level, which determines the path to the codebook of next level using partition tree that originally possesses the codeword for *X*. The encoded source bits are also determined by the codebook path and transmitted to the decoder. The total number of coset levels (and in turn the data rate) may vary depending on the distortion and noise that exist between the predictor and the received bitstream. Moreover, each coset level can be taken as a single level partition tree. In order to achieve the required rate for that level, a separate dedicated encoder and decoder can be designed. Although this slightly increases the complexity of the encoder, such a design allows the use of linear correction codes for each level and improves overall error resilience of the codec against channel and propagation errors.

### Pixel domain Wyner–Ziv (PDWZ) video coding architecture

**Fig. 8:**
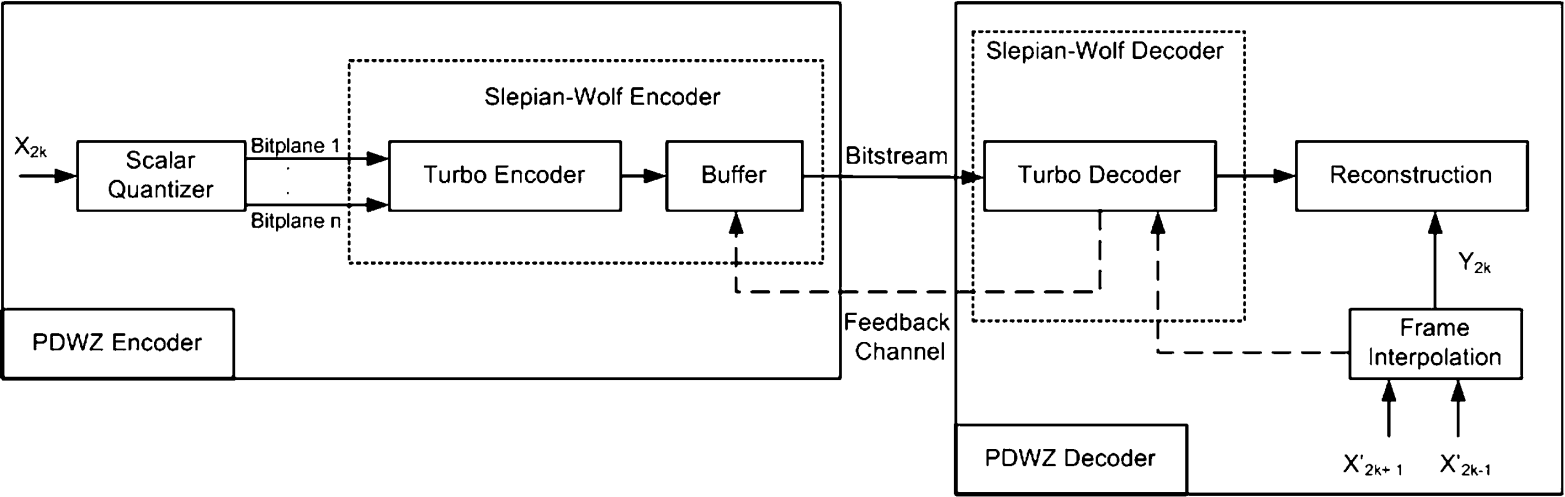
PDWZ architecture (Aaron et al. 2002)

The WZ architecture, also widely known as Stanford DVC architecture, was originally designed based on PDWZ (Aaron et al. 2002) coding. In later years, it was enhanced to TDWZ (Aaron et al. 2004b). In pixel domain WZ codec, which relies on intra-frame encoding and inter-frame decoding, the video sequence is spilt into *X* and $Y$, which represents the sets of even, and odd frames, respectively. Intra-frame encoding is used to encode *X*, given that *X* does not have any knowledge of $Y$. The redundancy between successive frames is exploited to determine the side information $(Y)$ at decoder, which in turn employs *Y* to conditionally decode $X$. The architecture of PDWZ codec is shown in Fig. 8.

The following discusses the encoder and decoder modules of the PDWZ codec:

#### Encoder

Let the frames to be encoded represented by *X*_1_, *X*_2_, …, *X*_*N*_, and the set of odd numbered frames $X_{2i + 1}$ termed as *key frames* are available at decoder, where $i \in \left\{ {0,1, \ldots ,\frac{N - 1}{2} } \right\}$. Therefore, steps for the compression of even numbered frames *X*_2*i*_ are as follows:

*Scalar quantizer* The symbol stream is initially generated by quantizing each pixel of every row of the entire frame at 2^*M*^ distinct levels using uniform scalar quantizer. The resulting quantized symbol stream of length $L$ for each even numbered frame is then fed into the Slepian–Wolf turbo encoder.

*Turbo coder and buffer* In order to achieve bit rate flexibility, rate compatible punctured turbo (RCPT) coding is implemented, which dynamically adapts to the coding parameters associated with mismatches that occur between the frame to be encoded and its side information. Each block of input symbols from the quantized stream is assigned a parity sequence and the blocks that have same parity sequence are grouped together in the same coset. Thereafter, the parity sequence is temporarily stored in buffer and transmitted in small chunks to the decoder as and when required. Such an arrangement ensures that the encoder will transmit only a small amount of parity bits to the decoder for reconstruction of quantized bitstream. However, the decoder continues to generate feedback requests until the quantized bitstream has been reconstructed with desired quality parameter.

#### Decoder

*Frame interpolation model and side information generation* Temporal interpolation between two successive key frames is performed to generate side information for the current frame to be decoded. However, the decoder design is flexible enough to adopt various interpolation techniques, ranging from simple average interpolation to complex symmetric motion vector (SVM) based motion compensation, which may include multiple frame predictors and intelligent segmentation features. The interpolation technique simply averages the pixel values of successive key frames to predict the pixel value of the non-key frame in between them at the corresponding location. However, SVM interpolates the motion based on the assumption that the motion vector remains the same between the successive key frames. Therefore, the block matching is performed between the successive key frames in order to estimate the symmetric motion vector for the given block of the sandwiched non-key frame. Next, the decoder performs statistical correlation between the frame to be decoded and the corresponding side information which is required for the conditional estimation of a given frame in the reconstruction module.

*Reconstruction* Each pixel of the frame can be reconstructed provided that its decoded bitstream and side information are available at the decoder. Since symbols are grouped together in cosets associated with the levels of quantisation, therefore if the side information is close enough to the reconstructed signal resulting from the decoded bitstream, it may fall within one of the coset‘s bins. Alternatively, the reconstruction process relies only on the signal to be reconstructed, quantizes it to the bin boundaries and ignores the side information. Such scenarios may happen when there are high motion frames and various occlusions in place.

The turbo decoder accompanied by the side information $(SI_{2i} )$ and the received parity bits generates an estimation of the quantized symbols (*q*) which produces an estimate $(u^{\prime})$ of the original pixel (*u*) for the given frame (*f*_2*i*_) using the following reconstruction function:

8 $$u^{\prime} = Estimate\left( {u|q,SI_{2i} } \right) = \left\{ { \begin{array}{*{20}l} {b_{l } , \quad v \le b_{l } } \\ {v, \quad b_{l } < v < b_{u } } \\ {b_{u } , \quad v \ge b_{u ,} } \\ \end{array} } \right.$$

where $v, b_{l }$ and *b*_*u*_ represents the pixel in $SI_{2i}$, upper, and lower boundaries of quantization bins indexed by 2^*M*^ = *q* levels of quantization, respectively. The above expression states that if a pixel values lies within the quantization index boundaries, then *u*′ extracts its value from *v*, otherwise it chooses one of the boundary values near *v*.

#### Enhancements to PDWZ

Several enhancements have been made to the original PDWZ codec design with a focus on modules such as rate-control, decoding algorithm, side information generation and correlation noise modelling. Table 1 summarizes the enhancements made to the aforementioned modules and their discussions from a number of well-known works in literature. Some of them are outlined as follows:

**Table 1:** Enhancements to PDWZ

Module	Summary	References
Rate-control	Encoder rate-control algorithm without feedback channel at the expense of increased encoder complexity An efficient block motion-estimation algorithm at encoder for estimating bitplane error probability and low complexity side information A code mode decision algorithm at encoder to improve coding performance	(Du and Shen 2009)
	Encoder based rate-allocation algorithm that computes the number of bits to encode each WZ frame without significantly increasing encoder complexity Uses a Laplacian random variable to represent the difference in bitplane values between the original frame and corresponding side information Defines a probability mass function to estimate the aforementioned random variable Estimates the bit error probability for each bitplane based on the error correcting capacity of the turbo code and frame rate of the video Prevents increase in distortion due to excessive errors in decoded bitplanes by discarding parity bits and sets decoded frame to side information if residual error probability estimated at decoder is above a given threshold	(Morbee et al. 2007)
Decoding algorithm	A mode decision scheme that can be executed at encoder or decoder (or both) to determine if the correlation noise estimation between a frame to be encoded and its side information is weak, and if block-based intra-frame coding should be selected instead of block-based WZ coding Shows that the relationship between the frame to be encoded and the side information at the decoder (defined as correlation noise statistics) is not spatially stationary Determines the selection criteria for mode decision by exploiting spatial and temporal statistics Creates a binary map whose entries indicate which blocks of a frame to be encoded should be intra- or WZ-coded. A simple entropy coding algorithm is used for efficient processing of this information	(Tagliasacchi et al. 2006a)
	A coding distortion model that can be used to determine the value of coding parameters such as quantization step size, target distortion, distortion predictions under certain coding constraints Selects the quantization step size of each video frame to meet the target distortion Shows that the accuracy of distortion predictions is limited by computation capacity of PDWZ encoders and the stability of distortion constraints	(Roca et al. 2007)
	A decoding algorithm based on turbo codes that requires a small subset of parity bits at the decoder for each WZ frame, and exploits the temporal correlation of the video sequence using previously reconstructed frame as noisy side information Mismatches between the side information and frame to be decoded are represented by pixel values and parity bits Uses a suboptimal approach to convert pixel values to soft information for the parity bits Uses hyper-trellis codes to combine multiple states of original trellis code Improves codec performance without increasing decoder’s complexity	(Avudainayagam et al. 2008)
	A decoding algorithm that incorporates side information computed from either lossless or quantized frames A mode decision scheme at the decoder (similar to the one in Fan et al. 2010) is used to improve the correlation noise statistics	(Trapanese et al. 2005a)
Side information	An extrapolation module to generate side information based on motion field smoothening filter to enhance performance of a low-delay PDWZ codec Uses overlapped motion estimation, in particular motion field smoothening filtering and spatial-interpolation for un-overlapped regions	(Natário et al. 2005)
	Encoder based motion-compensation module that sends hash codewords of the frame to be decoded to the decoder Hash codewords complements the side information and lead to efficient frame reconstruction Enables low-complexity encoding while maintaining high compression ratio	(Aaron et al. 2004a)
Correlation noise modeling	A correlation noise estimation module that performs online estimation of the error distribution at the decoder A temporal model that estimates correlation between frames under different levels of granularity such as: frame, block and pixel levels An improved rate-distortion performance at lower granularity level Collects correlation noise statistics locally on a block-by-block basis and at pixel level	(Brites et al. 2006)
	An enhanced correlation noise model with reasonable coding efficiency gain Shows that a Laplacian model is not an optimal choice to represent the distribution of correlation noise, since the rate at which the tails of the model decreases to zero is slower than the empirical distribution (see Figure 3 of Macchiavello et al. 2007) Presents improved modeling of the tails for the turbo decoding process A turbo decoder that assigns a higher likelihood to estimated values far apart from the corresponding side information to increase the chance of decoding outliers and enhance the reconstruction quality	(Trapanese et al. 2005b)

- Hyper-Trellis decoding for PDWZ video coding is proposed in (Avudainayagam et al. 2008) to optimize the approach for the reconstruction of WZ frames. A new decoding algorithm is presented which encapsulates and combines various states of the original trellis code. The results show that the proposed approach not only reduces the complexity of the decoder, but also increases the reconstruction quality by 9–10 dB.
- In contrast to the original PDWZ codec design, where decoder controls the encoding rate via feedback channel, a low complexity rate-control algorithm that executes at the encoder is proposed in (Du and Shen 2009; Morbee et al. 2007). The proposed design shifts the rate-control functionality from decoder to encoder, and eliminates the feedback channel, which not only reduces the decoding complexity, but also the delays.
- A distortion control algorithm is presented in (Roca et al. 2007) to overcome the coding distortions in PDWZ codec. The algorithm helps in choosing the optimal steps size for quantization levels associated with certain target quality. However, the experimental results showed that the accuracy in prediction of distortion function is primarily dependent on the encoder’s computational power.
- In (Natário et al. 2005), an algorithm to refine side information generation module is proposed which utilizes an intra-frame encoding/inter-frame decoding architecture. In addition, the complexity of the decoder can be further reduced if the key frames (used to generate side information) are extrapolated from previously decoded frames. The extrapolation module exploits the motion field smoothening filter for efficient and accurate reconstruction of side information.

### Transform domain Wyner–Ziv (TDWZ) video coding architecture

**Fig. 9:**
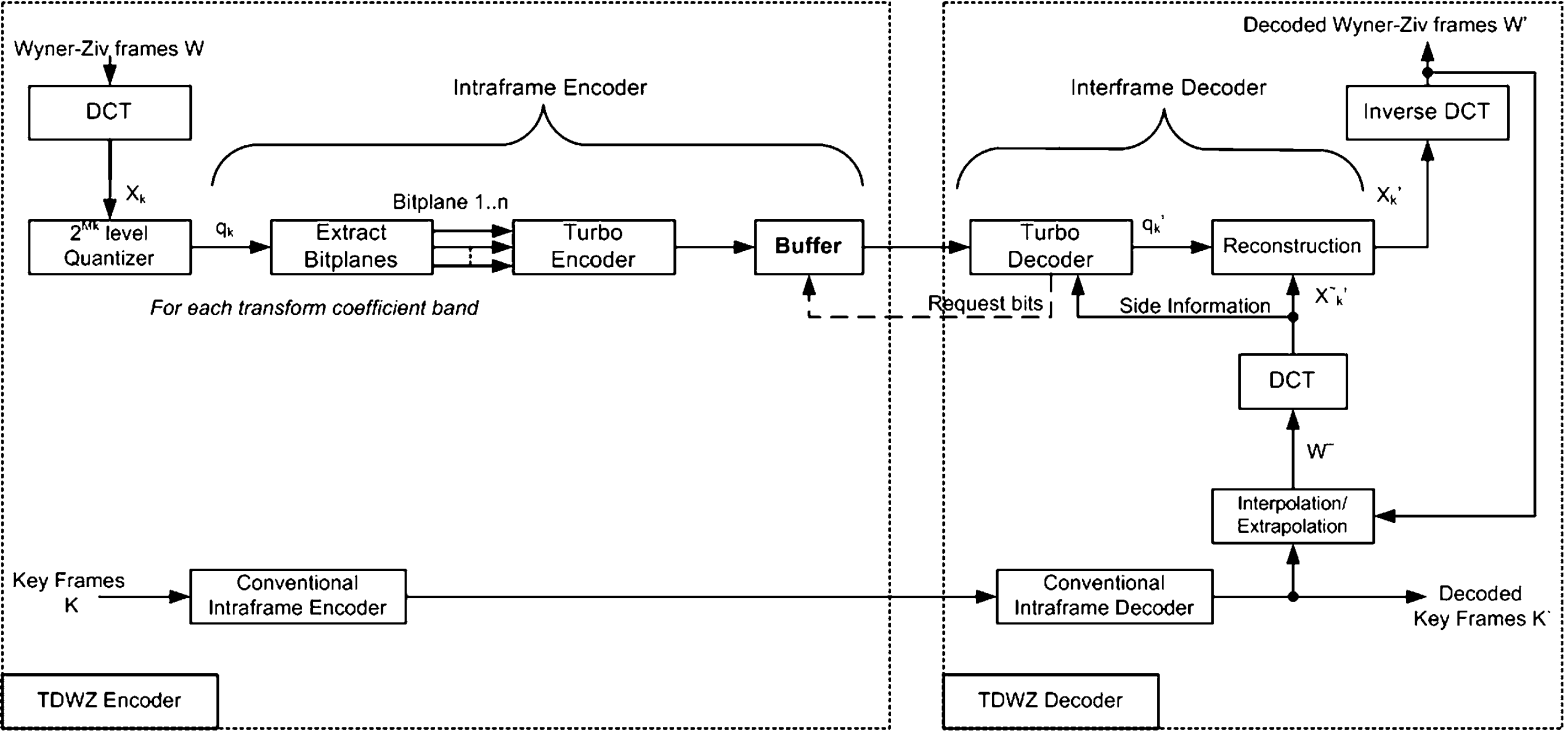
TDWZ architecture (Aaron et al. 2004b)

The pixel domain WZ codec has been extended from pixel-domain (Aaron et al. 2002) to transform-domain (Aaron et al. 2004b), which exploits spatial correlation within a given frame and temporal correlation among adjacent frames to achieve better rate-distortion performance as shown in Fig. 9. The inclusion of DCT module makes TDWZ a more practical WZ codec, which encodes key and WZ frames using conventional intra-frame encoder, and WZ encoder, respectively. At decoder site, key frames are reconstructed via conventional inter-frame decoder, whereas decoding a WZ frame requires side information generated by the previously decoded key frames and WZ frames. In the following sub-sections, we will discuss only those modules of TDWZ which differ from those in the PDWZ codec architecture.

#### Encoder

The TDWZ codec splits the video sequence into key frames and WZ frames encapsulated within a group of pictures (GOP).

*Discrete cosine transform (DCT)* Each WZ frame is decomposed into sub-blocks which undergo DCT transformation and generate DCT coefficients. These DCT coefficients are assigned to different bands according to their position in the DCT block. Thereafter, each DCT band is quantized into a number of quantization levels via a uniform scalar quantizer.

*Bit*-*plane extraction* Quantized DCT coefficients (symbols) are grouped together into bit-plane vectors and fed independently to the Slepian–wolf turbo encoder.

*Turbo encoding* Turbo encoder starts encoding each bit-plane vector using rate-compatible punctured turbo (RCPT) codes (Qing et al. 2007), and the resulting parity information is temporarily stored in buffer and subsequently transmitted to the decoder in small chunks upon receiving requests from the feedback channel.

*Conventional intra*-*frame encoder* Intra-frame encoding mode of conventional video codecs such as H.264/AVC is used to encode key frames, which upon being received at decoder, are reconstructed via conventional Intra decoder.

#### Decoder

The decoder processes the video frames according to the GOP configuration and operates conventional intra decoder and WZ decoder in parallel for the reconstruction of key frames, and WZ frames respectively. However, in (Aaron et al. 2004b), the GOP size was set to 2, which implies that every alternate frame is a key frame. In later versions (e.g. Kubasov et al. 2007b; Macchiavello et al. 2009a), the decoder was extended to support GOP sizes of 4, 8 and 16 frames.

*Conventional intra*-*frame decoder and frame interpolation/extrapolation* Key frame decoding is relatively straight forward, since it only exploits the spatial correlation in the given frame. However, the reconstructed key frame also provides an estimate for the WZ frame to be decoded. Frame interpolation/extrapolation is performed on each decoded current key frame along with previously reconstructed frames to produce the side information $W^{\sim }$ required for reconstructing the WZ frames.

*DCT transformation* On receiving side information $W^{\sim }$, block-based DCT is performed and the resulting transformed coefficients are aligned to form coefficient bands $X_{k}^{\sim }$, which is an estimate of each decoded bitplane of the received WZ frame $X_{k}$.

*Turbo decoding and reconstruction* The turbo encoder-decoder in PDWZ and TDWZ is utilized as a Slepian–Wolf codec. Each bit-plane vector is turbo decoded, given that the side information $X_{k}^{\sim }$ and the residual statistics are available. However, if the decoder cannot decode a bit-plane, it requests additional parity bits from encoder via feedback channel, and the process continues until a certain acceptable level of bit error rate performance is achieved.

#### Distributed coding for video services (DISCOVER)

DISCOVER (2005) is an European video coding project, which has introduced several new modules and improved existing modules to enhance the overall performance of the TDWZ codec. Notably:

1. Adaptive GOP selection and encoder rate-control mechanisms with input from decoder‘s virtual channel are introduced;
2. Turbo coder is replaced by LDPC coder (Varodayan et al. 2006);
3. Correlation noise modeling is performed between the side information and corresponding WZ frame via soft input computation to enhance the reconstruction quality.

**Fig. 10:**
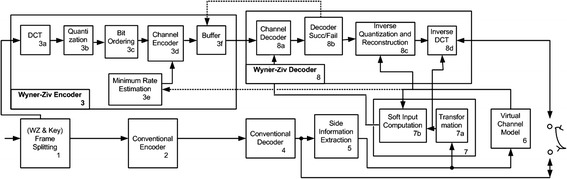
DISCOVER architecture (Artigas et al. 2007)

The architecture of DISCOVER is shown in Fig. 10. Blocks 1–3 represent the encoding phase, which splits the incoming video sequence into two parts for encoding as key frames and WZ frames, using conventional encoder, and WZ encoder, respectively. Blocks 4–8 represent the decoding phase, which decodes the key frames and generates side information from which coefficients of WZ frames are estimated and then applied for decoding the WZ frames. If decoding fails, further information (e.g. more parity bits) may be requested by the decoder from the encoder through a feedback channel illustrated by the dotted line. This repeats until decoding is successful and the final video is reconstructed by multiplexing the decoded key frames and WZ frames. The following further elaborates on the aforementioned key enhancements.

The selection of GOP size is made adaptive to varying temporal correlation in the video sequence (Ascenso et al. 2006). By analyzing the video frames, larger or smaller GOP size can be employed for sequences having higher, or lower temporal correlation among frames, respectively. A hierarchical clustering algorithm executed at the encoder to group frames of similar motion activity, is responsible for making the decision about the GOP size. It is observed that the codec exhibits better rate-distortion performance when such adaptive GOP is employed as compared to using fixed GOP.

The LDPC channel codes introduced in (Varodayan et al. 2006) have replaced the turbo channel codes not only in DISCOVER, but almost all TDWZ architectures. The LDPC encoder is comprised of syndrome generator and the accumulator which stores the syndrome bits generated from LDPC codes to form the accumulated syndromes for transmission to the decoder. In contrast to turbo codes, LDPC codes efficiently utilize the capacity of channels under varying communication requirements. Furthermore, a CRC sum of the encoded bit-plane is transmitted to the decoder to perform error checking of its received bits.

The rate-distortion approach proposed in (Wyner and Ziv 1976) is used to develop the rate-control module at the encoder. It computes the minimum rate employed by the source for a given distortion measure, and enables the encoder to determine the minimum number of accumulated syndrome bits to be transmitted per bitplane for each coefficient band. This enables the DISCOVER to exhibit comparable rate-distortion performance with conventional H.264 Intra codecs. To model the correlation noise between the transform bands of WZ frame and corresponding side information, DISCOVER uses the Laplacian error distribution (Girod et al. 2005; Brites et al. 2006), which considers the variance in noise statistics pertinent to spatial and temporal correlation and evaluates the distribution parameter online. The resulting noise correlation model aids in transforming the side information’s transform coefficients into soft-input for the LDPC decoder.

#### TDWZ based on discrete wavelet transform (DWT)

An alternative transformation technique to DCT in use by some TDWZ architectures is DWT, which decomposes a video frame into a set of wavelets with different locations (shift) and scales (resolution). Any decomposition involves a pair of high or low frequency components (sub-bands) that correspond to the detailed parts, and smooth parts, of the video frame, respectively. The wavelets are represented as coefficients which are organized into multi-resolution levels. The main advantages of DWT over DCT are the absence of blocking artefacts as it works on the whole frame rather than on separate blocks, better de-correlation (redundancy removal) property, and inherent scalability. However, popular wavelet coding schemes such as Embedded Zerotree Wavelet (EZW) and Set Partitioning In Hierarchical Trees (SPIHT) produce code words with variable length, which can make the compressed video streams very susceptible to transmission errors in wireless channels. The effects of these errors can in turn propagate to the entire frame during the video reconstruction (Xue et al. 2010).

Bernardini et al. (2006) proposed a wavelet domain WZ codec, which shares a similar architecture as TDWZ, but the DCT/Inverse DCT modules are replaced by their DWT equivalent. In addition, a *Motion Evaluation* module is introduced at the encoder to predict the quality of side-information that will be generated by the decoder based on the amount of motion between successive frames. This is to enable local rate estimation by the encoder and eliminate the need for a feedback channel. However, in WVSN, the quality of side-information does not depend only on the frame motion, but also on the conditions of the channel through which the bits are received and used for constructing the side-information. The authors also introduced a *Modulo Reduction* module at the encoder to reduce the range of the wavelet coefficients so that they can be quantized and coded more efficiently.

Similarly, Guo et al. (2006) presented a wavelet-based TDWZ codec in which high-order statistical correlation among wavelet coefficients is exploited to improve the coding efficiency of WZ frames. The authors introduced an *Entropy Reorder* module at the encoder to reorganize quantized wavelet coefficients into a tree structure using an algorithm based on ZeroTree Entropy (ZTE) coding. The significant coefficients are turbo coded and the parity bits are transmitted. The significance map is intra-coded by an *Entropy Coding* module and transmitted to the decoder, which uses it to extract the significant coefficients of the side-information for reconstructing the WZ frames.

So far the new features introduced above are mainly at the encoder side. For the decoder side, Liu et al. (2010) proposed a wavelet-based TDWZ codec with improved side-information estimation based on multi-resolution motion refinement (MRMR). The main idea is for the decoder to progressively learn from already-decoded lower-resolution data to refine the motion estimation, which in turn improves side-information quality and coding efficiency for higher resolution data. In order to achieve this, two features are introduced: (1) an *Over*-*Complete DWT* module to transform a frame into subbands in an over-complete form to overcome the shift-variance problem of critically sampled DWT; (2) a *Motion Refinement* module to refine the motion vector by motion estimation between current and previous low frequency subbands, and use the refined motion vector to generate side-information of higher-frequency subbands at the same decomposition level. This process repeats until all levels are decoded.

#### Enhancements to TDWZ

Similar to PDWZ, several enhancements have been made to the original TDWZ codec design with a focus on modules such as rate-control, decoding algorithm, side information generation, and correlation noise modelling. A table summarizing the enhancements made to TDWZ and their discussions are shown in Table 2. Some of them are outlined as follows:

**Table 2:** Enhancements to TDWZ

Module	Summary	References
Rate-control	Rate-allocation scheme without using feedback channel and without significantly increasing the encoder complexity A linear rate estimation model that avoids over or under estimation and achieves optimal rate-distortion performance Shows a reasonably good encoder rate allocation performance while maintaining coding efficiency	(Sheng et al. 2010)
	Encoder based rate-allocation scheme that predicts the number of encoding bits as a function of the coding mode and quantization parameters Predictions select the best coding mode and quantization parameters for encoding WZ frame without significantly increasing encoder’s complexity Shows relatively low loss in rate-distortion performance as compared to conventional decoder based rate allocation scheme	(Sheng et al. 2008)
	The DISCOVER codec generates the band of transform coefficients after performing block-based transformation and quantization on each WZ frame An improved rate-control algorithm that computes the initial number of bits to transmit for each bitplane and band Does not require excessive number of iterations, hence improves the coding efficiency	(Artigas et al. 2007)
	A feedback channel-driven rate-control codec with improved modules using special coding tools An improved motion learning algorithm with reasonably good rate-distortion performance	(Martins et al. 2010)
	A hybrid procedure for rate estimation at encoder side for wavelet based WZ codec with no feedback channel Quantized high resolution subbands entropy coded using low-complexity intra-coding to avoid under-estimation of required bit rate For lower resolution subbands, the entropy of the bitplane crossover probability is used as an estimate	(Bernardini et al. 2011)
	Channel adaptive rate control for feedback-free wavelet-based WZ; priority given to lower frequency subbands that hold more information about a frame than higher frequency subbands	(Rui et al. 2013)
Decoding algorithm	A decoding algorithm that exploits previously reconstructed transform bands to reduce the total number of bits needed for reconstructing the remaining bands	(Martins et al. 2010)
	Presents TRACE (TRansform domain Adaptive Correlation Estimation) for WZ decoder Progressively learns the correlation among frames during the frame reconstruction process A convex optimization based band-level correlation estimation method that minimizes the theoretical required bit rate	(Fan et al. 2010)
	A multi-hypothesis based WZ decoder that exploits the redundancy between multiple side information(s) and the source frame Uses both block-based and optical flow-based side information generation methods to generate multiple side information(s) An optical flow based frame interpolation algorithm to compensate side information estimation weaknesses in block based methods Employs multiple soft-inputs for decoding and reconstruction based on a weighted joint distribution, which reduces the required bitrate and improves the quality of reconstructed frames	(Huang et al. 2011)
	A bit-level context-adaptive correlation model for the decoder of a wavelet-based WZ codec with MRMR (Liu et al. 2010) to achieve better prediction of the bit probability distribution.	(Qing and Zeng 2014)
Side information	An improved side information refinement framework that utilizes both spatio-temporal correlation and previously reconstructed transform bands Uses a non-local means denoising process that exploits partially decoded side information(s) progressively available at the decoder A denoising process that progressively regenerates side information with improved quality	(Shen et al. 2012)
	A side information generation algorithm based on semi super-resolution frame that exploits past and future reference frames for block-based motion estimation process Iteratively generates side information using channel decoder following the decoding of a low resolution base layer to produce high quality decoded frame Low resolution encoding reduces the encoding complexity Encodes the residual WZ frame by cosets	(Macchiavello et al. 2007)
	An extrinsic information transfer (EXIT) chart analysis for minimizing the mutual information variation in iterative low density parity check (LDPC) decoding during side information refinement process Obtains relatively good reconstruction quality with low coding rates	(Wen et al. 2012)
Correlation noise modeling	A correlation noise estimation algorithm that exploits the adjacent key frames to predict the quantization step of the quantization module Indices generated for two frames differ by smallest number of bits possible Shows improved WZ encoding bitrate for a given target quality as compared to the traditional coding schemes	(Micallef et al. 2012)
	An online approach for modelling correlation noise model parameters at the decoder Determines the temporal correlation between frames at various levels of granularity (DCT bands, DCT coefficients) Shows good rate distortion performance at higher estimation granularity	(Brites and Pereira 2008)
	A correlation noise model that utilizes cross-band correlation to precisely estimate the Laplacian parameters Uses a category map based on previously reconstructed bands to classify transformed coefficients of the current band Allocates each transformed coefficient to a Laplacian parameter based on its category More precise estimation of correlation noise model parameters leads to better rate-distortion performance	(Huang and Forchhammer 2009)

- It is observed that the accuracy of side information generation has a significant impact on codec’s overall performance. Several enhancements have been suggested for the estimation of side information in TDWZ codec. This includes a progressive side information refinement framework introduced in (Shen et al. 2012), which exploits the spatial and temporal correlation among previously decoded frames to gradually improve the side information as the decoding process progresses. Various approaches for the enhanced side information module based on progressive side information refinement, motion searching, resolution-progressive decoding, and extensive motion exploration are presented in (Macchiavello et al. 2007, 2009a, b; Liu et al. 2009; Wen et al. 2012). Research has also been done to transmit the hash signature of each bit-plane to detect and remove any errors during transmission to further enhance the side information module. Reasonable gains in overall codec’s performance have been reported at the expense of some additional complexity at the encoder (Aaron et al. 2004a; Ascenso and Pereira 2007).
- One of the most challenging tasks of the TDWZ codec is to assign an optimal number of bits to encode the WZ frame. Typically, a feedback channel is employed to inform the encoder about the required encoding rate. Since the encoder itself does not have access to motion compensation information of the WZ frame, significant degradation in rate-distortion performance may occur if insufficient bits have been allocated to the frame. However, for applications that only transmit data in one way such as broadcasting, employing a feedback channel is not possible. Sheng et al. (2008, 2010) proposed a rate-distortion algorithm for TDWZ in which the encoder estimates the number of bits per bitplane for each coefficient band as a function of quantization parameters and coding mode without using feedback channel to reduce the latency and complexity of the decoder site.
- Micallef et al. (2012) presented an algorithm to reduce the correlation noise between the bitplanes of WZ frame and the corresponding side information. The algorithm makes use of previously reconstructed key frames to estimate the quantization steps and configure the quantization values such that the mismatches between current WZ frame and its side information is reduced to a minimum. The proposed method is shown to provide significant performance gain as compared to traditional TDWZ codec.

## Comparison and analysis

This section compares and analyzes the reported functional aspects and performance involving the three video coding architectures discussed in “DVC architectures”.

**Fig. 11:**
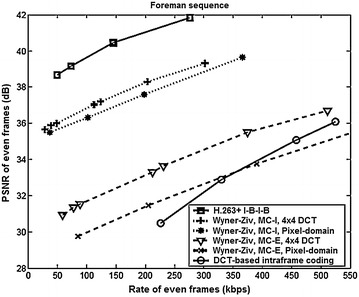
Rate-distortion comparison of PDWZ, TDWZ and H.263+ video coding architectures—foreman sequence (Aaron et al. 2004b)

The rate-distortion comparison among PDWZ, TDWZ and H.263+ video coding architectures reported in (Aaron et al. 2004b) for *Foreman* sequence is shown in Fig. 11, where MC-1 and MC-E represents motion-compensation using frame *Interpolation*, and *Extrapolation*, respectively. The first 100 even frames of *Foreman* QCIF sequences were used to evaluate the rate-distortion performance of the luminance component encoded at the frame rate of 15 frames per second. The TDWZ results were also compared with (a) DCT-based intraframe coding where the even frames are encoded as I frames; (b) H.263+ interframe coding with an I-B-I-B predictive structure; and (c) PDWZ codec.

The evaluation assumed the same rate and quality for the odd frames of all the schemes, and therefore the rate-distortion performances of odd frames were not included in the plots. It is evident from Fig. 11 that when highly reliable side information (MC-I) is used, the TDWZ codec is 7–8 dB better than the PDWZ codec. On the other hand, using less reliable side information (MC-E), the TDWZ codec yields a PSNR gain of 1–3 dB against DCT-based intraframe coding. It is also observed that compression efficiency loss is approximately 5 dB higher since the motion and occlusions in the foreman sequence make it more difficult to extrapolate the succeeding frames.

**Fig. 12:**
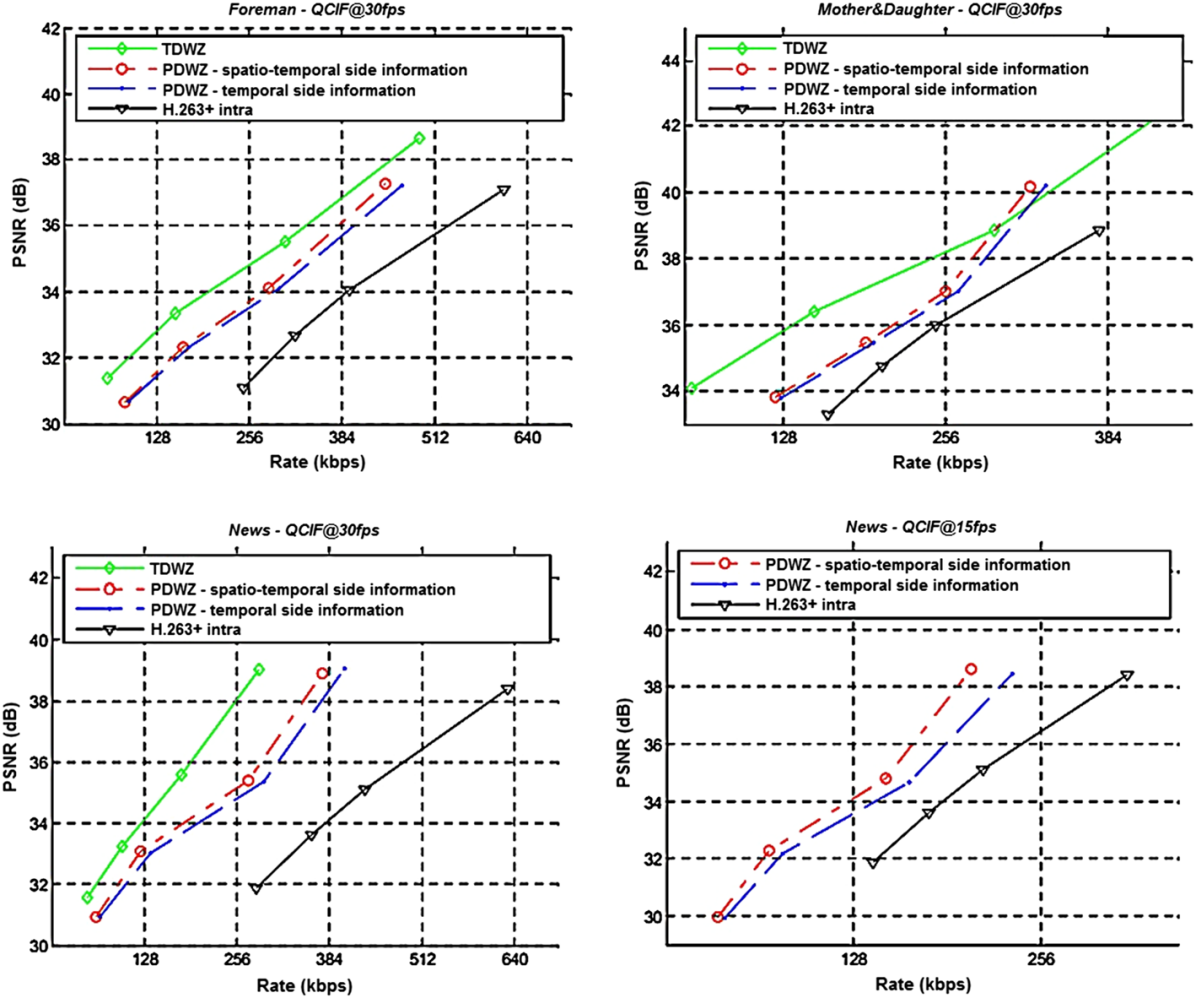
Rate-distortion curves of TDWZ, PDWZ, enhanced PDWZ, and H.263+ video coding architectures—different types of video sequence (Tagliasacchi et al. 2006b)

Tagliasacchi et al. (2006b) also proposed an algorithm that was integrated into the PDWZ codec and exploited its spatial redundancy without introducing any transform at the encoder side, thereby keeping the complexity of the encoding as low as possible. They presented the rate-distortion curve (Fig. 12) which showed noticeable improvement in the side information quality and rate-distortion performance. However, results may vary depending on the type of video sequence.

Experiments were carried out on Foreman, Mother and Daughter, and News video sequences using frame rate of 15 fps. From Fig. 12, it is evident that the rate-distortion curve for the TDWZ codec almost always outperforms PDWZ codec, even when the spatio-temporal side information is being used. On the other hand, the enhanced PDWZ design (Tagliasacchi et al. 2006b) improves the side information with spatial data without adding further complexity to the encoder, which bridges the gap between the original PDWZ and TDWZ codecs.

The DISCOVER and PRISM architectures were compared to conventional H.264/AVC codec in (Tonoli et al. 2009), under a transmission scenario over a network with and without channel errors. The DISCOVER codec has been configured with GOP size 2 under the assumption that lost packets will be requested through the feedback channel, which avoids a deterioration of the frames’ reconstruction quality with potential cost of a higher bit rate requirement. The H.264/AVC uses the same key frame setup of the DISCOVER, which transmits each slice of a given frame in a single packet with GOP size 2 in IBIB^3^ mode. The encoded bitstreams were transmitted over a network with a packet loss ratio (PLR) of 0 or 5 %, and the PSNR for foremen sequence was evaluated and analyzed on a frame-by-frame basis.

**Fig. 13:**
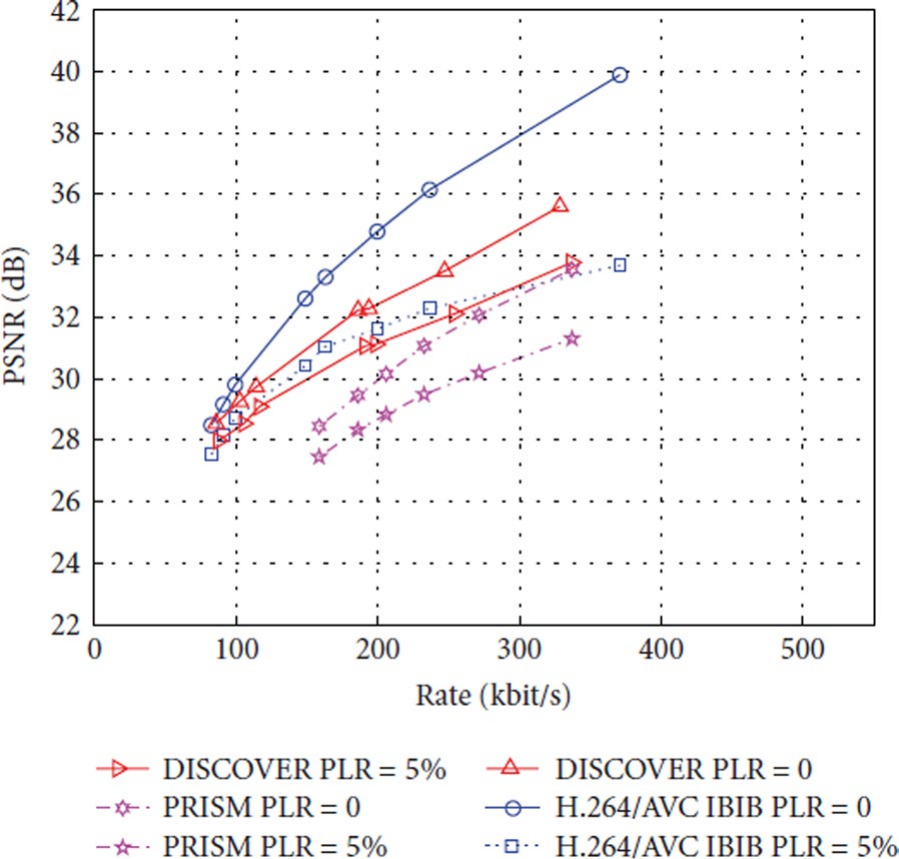
Rate-distortion comparison of DISCOVER, PRISM and H.264 video coding architectures—foreman sequence (Tonoli et al. 2009)

As shown in Fig. 13, it can be observed that DISCOVER generally outperformed PRISM in terms of PSNR in both error-free case (PLR = 0) and in the presence of channel errors (PLR = 5 %). However, the performance in terms of both compression and error resilience depends upon the content of the test sequence. In contrast to H.264/AVC, the DISCOVER tends to offer better PSNR performance as PLR increases. This is due to the reason that H.264/AVC is not able to compensate for lost information, whereas the DISCOVER can request for additional parity bits through the feedback channel. As a result of such architectural differences, it may not be considered fair to compare their performances.

In the PRISM codec, the visual quality is more uniform than in DISCOVER, because every frame is encoded by combining traditional and WZ encoding in a block wise fashion. However, this uniformity in the perceived quality carries the drawback of an increased sensitivity to drift errors with respect to the DISCOVER architecture. Given that PRISIM is unable to handle drift errors in a satisfactory way, even if the average quality of the video sequence is fine, the presence of a few regions in the frame that contain drift errors could still be visible to the human eye.

Table 3 summarizes a functional comparison of the three primary DVC architectures. The following compares these key functional features in more detail:

**Table 3:** Functional comparison of primary DVC architectures

Features	Architectures		
	PRISM	PDWZ	TDWZ
Video coding unit			
Block-based coding	✓		
Frame-based coding		✓	✓
Rate control			
Encoder rate control	✓		✓*
Decoder rate control		✓	✓
Channel coding			
BCH code	✓		
Turbo code		✓	✓
LDPC code		✓	✓

* In (Kubasov et al. 2007b), a hybrid encoder-decoder rate-control mechanism for TDWZ is proposed

- *Block*-*based and frame*-*based coding* For exploiting spatial correlation to generate bitstream, various DVC architectures employ either a block-based or a frame-based encoding approach, each having its own salient characteristics. Block-based coding is more adaptive to spatial variations, thus exploits local features of a video frame more efficiently and has a better reconstruction quality at the decoder side. On the other hand, frame-based coding has the benefit of being able to deal with larger datasets, and its comparatively lower complexity is a desirable feature for more efficient channel coding. During intermediate processing stages, although a frame-based coding approach may still offer the advantage of block-based coding, the final bitstream is associated with the entire frame rather than providing support for smaller spatial blocks. Berkeley’s PRISM architecture and Stanford’s Wyner–Ziv architecture are examples of block-based, and frame-based coding approaches, respectively.
- *Encoder/decoder based rate*-*control* One of the challenging tasks for DVC architecture is to allocate the number of bits associated with each frame/block for transmission to the decoder. In some architectures, this responsibility is placed either on encoder or decoder (or both encoder and decoder). If the decoder is involved in taking a decision about the encoding rate, feedback channel is required to provide a more sophisticated and tighter control over the number of bits transmitted from the encoder to decoder. Decoder performs complex processing during frame reconstruction, and depending upon the requirements to achieve predetermined target quality, the decoder may request the encoder to change its encoding rate. For example, parity bits transmitted from the encoder are analyzed by the decoder to determine whether these bits are insufficient to achieve an acceptable level of performance. If not, it will request additional parity bits from encoder, rather than using the decoded frames with well-built traces.

Such approach is applicable to real-time video coding (active communication mode) applications because the decoder can estimate the number of additional bits required only at the time of decoding. Feedback channel also has an impact on decoder’s complexity and may introduce latency if used frequently. An improvement to the side information generation module may involve restricting the number of feedback requests from the decoder. Alternatively, the encoder rate-control approach eliminates the need for a feedback channel (passive communication mode) and estimates the number of bits needed to achieve the desired target quality with additional complexity at the encoder side. Berkeley’s PRISM architecture, and Stanford’s Wyner–Ziv architecture, are examples of encoder, and decoder based rate-control approaches, respectively.

A number of hybrid approaches have also been proposed as extensions of the Wyner–Ziv architecture in which the encoder estimates the number of bits, thereby limiting the use of feedback channel and reduce the overall latency (Kubasov et al. 2007b).

- *BCH, turbo and LDPC channel codes* BCH channel codes with ability to correct multiple bit errors are very simple and have tighter control on symbol errors during the channel coding process. Decoding of BCH requires performing simple algebraic operations (Bose and Ray-Chaudhuri 1960). BCH encoding and decoding in video coding architecture is also widely known as syndrome coding and decoding, respectively. Turbo codes, on the other hand, are forward error correction codes that can achieve a channel capacity comparable to theoretical bounds. They guarantee reliable communication even in the presence of noise and under bandwidth or delay constraints (Berrou et al. 1993). Finally, LDPC channel codes, which are the most sophisticated among the three, are the linear error correction codes specifically designed to transmit information under noisy channel conditions. Similar to turbo codes, they can achieve a channel capacity close to the theoretical maximum with a very low bit error rate. LDPC decoding time is dependent on the information block length (Gallager 1963). Berkeley’s PRISM and early Stanford’s Wyner–Ziv architectures employ BCH and turbo channel codes, respectively. However, in later years, LDPC replaced the turbo codes in both Stanford’s pixel and transform domain WZ architectures.

In addition to the aforementioned factors considered for functional comparison of DVC architectures, there are still other issues not sufficiently addressed in the existing literature:

- *Chroma components coding* In all three architectures including their enhancements, there is no mentioning of encoding and decoding procedures for Chroma component. Moreover, the performance comparisons have been done only for Luma components. Considering the significance of Luma component, more details on Luma coding in terms of compression mechanism as well as its impact on overall performance, should be provided.
- *Standardization* To date, there is still a lack of a standard implementation of DVC architectures. There exist a number of different implementations. For the sake of interoperability, standardization is an issue that needs to be addressed for practical and commercial adoption of DVC.

- *Compressed video transport bitstream* In literature, for all the WZ architectures (pixels/transform domain), the WZ bitstream and H.264 (or conventional Intra coder) bitstream are sent separately to the decoder. However, for practical applications, a single transport bitstream that combines the compressed WZ and H.264 frames is required.

Moreover, regarding DVC application to WVSN, there are still challenges to be overcome in order to enable the delivery of high quality video stream, such as:

- *Latency* The conventional DVC codecs ideally assumes the block interleaver size in Slepian–Wolf turbo code is large enough to encode an entire WZ frame, which is unrealistic for practical use since it will add significant computational delay to the end-to-end system. Unlike the WVSN requirement, the system will be unable to respond with a timely WZ decoded output due to the intense Slepian–Wolf turbo coding and decoding delay.
- *Bitrate allocation* In most of the DVC codecs (PDWZ/TDWZ), the bitrate of WZ frame is determined by exploiting the statistical correlation between the side information and original WZ frame. Due to the architectural constraint, the encoder does not have access to side information. Therefore, the accurate estimation of the number of parity bits that will be sufficient for decoding is very complex. This rate control problem is commonly addressed by using the feedback channel between encoder and decoder. In case of decoding failure, a request for more parity bits will be sent to encoder via feedback channel. However, the request is unable to inform the encoder the number of parity bits that should be sent. Sending redundant parity bit increases the transmission energy consumption, a situation that will be exacerbated in multihop WVSN scenario. On the other hand, architectures without feedback channel usually compute the side-information at encoder to predict the bitrate, which results in greater computational complexity (and associated energy consumption) at the encoder that consequently decreases the lifetime of the source node and the entire network.

In our work presented in (Imran et al. 2012), we also evaluated and analyzed the performance of DVC architectures against conventional PVC-based codec for multihop WVSNs to provide an insight about the computational (encoding/decoding) complexity, energy consumption, node and network lifetime, processing and memory requirements, and the quality of reconstruction between them. Our findings revealed that DISCOVER (a transform-domain Wyner–Ziv Codec) is the most energy-efficient encoder for single-hop WVSN environment. However, considering its dependency on feedback channel for decoding operation and higher communication energy, it is only suitable for real-time applications. However, we also discussed other feedback-less DVC variants that can be used in both real-time and non-real time applications with comparable energy requirements as DISCOVER. H.264 Intra is found to have lower communication energy consumption than DVC codecs due to its better compression ratio. However, it consumes higher computation energy due to its greater encoding complexity, which significantly increases its overall energy consumption (Imran et al. 2012).

We also investigated the lifetime of both source and relay nodes and the overall network for each of the video codecs. The relative order of the codecs in terms of the lifetime of the source node, which is considerably shorter than that of relay node and thus dictates the network lifetime, expectedly follows the computational energy results.

Further findings on processing and memory requirements supported our assessment that DVC-based codecs outperform the conventional codec. The variation in the processing and memory requirements between the DVC-based codecs is primarily due to the GOP difference and the compression technique.

## Conclusion

In this paper, we reviewed and synthesized promising DVC architectures and the enhancements made to them in recent years. In addition, the significance of DVC in the evolving WSN application domain is discussed. Future research directions for DVC may include enhanced side information generation, rate-control, correlation noise modelling, as well as the design of novel and efficient channel codes. Enhancing target reconstruction quality, enabling flexible complexity distribution between encoder and decoder, and multi-view DVC coding are still open research issues that should be investigated.

## Abbreviations

AVC: advanced video coding
CRC: cyclic redundancy check
DCT: discrete cosine transform
DISCOVER: distributed coding for video services
DVC: distributed video coding
DWT: discrete wavelet transform
EXIT: extrinsic information transfer
EZW: embedded zerotree wavelet
GOP: group of pictures
LDPC: low density parity check
MPEG: moving pictures experts group
MRMR: multi-resolution motion refinement
PLR: packet loss ratio
PSNR: peak signal-to-noise ratio
PVC: predictive video coding
PDWZ: pixel domain Wyner–Ziv
QCIF: quarter common intermediate format
QoS: quality of service
RCPT: rate compatible punctured turbo
SPIHT: set partitioning in hierarchical trees
SVM: symmetric motion vector
TDWZ: transform domain Wyner–Ziv
TRACE: transform domain adaptive correlation estimation
VoD: video-on-demand
WSN: wireless sensor network
WVSN: wireless video sensor network
WZ: Wyner–Ziv
ZTE: zerotree entropy

## Footnotes

1 Entropy coding determines the minimum bits required to represent one source symbol. The transmission channel is assumed to be error free unless otherwise specified.

2 BCH error correcting codes proposed by Bose, Ray-Chaudhuri, and Hocquenghem in 1960. The abbreviation comprises of the initials of the three inventors.

3 With GOP size 2, IBIB mode uses bidirectional predicted frame after every alternate Intra frame sequence.
